# Impact of internal identity asymmetry on employee's behaviors and feelings: A mediating role of psychological distress

**DOI:** 10.1016/j.heliyon.2024.e31438

**Published:** 2024-05-17

**Authors:** Rida Batool, Qingfeng Tian, Erhua Zhou, Najmul Hasan

**Affiliations:** a School of Management, Northwestern Polytechnical University, Xi'an, China; b School of Management, Huazhong University of Science & Technology, Wuhan, China; c BRAC Business School, BRAC University, Dhaka, Bangladesh

**Keywords:** Internal identity asymmetry, Psychological distress, Coping strategies, Behavior, Feelings

## Abstract

Individuals may experience internal identity asymmetry when they feel misidentified and believe their colleagues do not recognize their work-related identities. This research examines the impact of internal identity asymmetry on their behavior and emotional outcomes at the workplace in Pakistan. Data was collected through a survey and received responses from 393 participants at different levels of management in various sectors of Pakistan. A partial least square-based structural equation modeling technique has been used to validate the proposed research model and develop hypotheses. The findings indicate that psychological distress has a positive indirect effect on both outcomes, such as individual work performance and well-being during personal and professional base asymmetries time. The result indicates that employees face psychological distress while experiencing internal identity asymmetries, which may decrease the performance and well-being of the employees. Findings highlight the importance of coping strategies in improving the performance and well-being of employees. Managers can be supportive in maintaining a positive workplace environment where individuals can have a more accurate self-perception and a better understanding of their colleagues' perspectives. This, in turn, enables them to adopt appropriate coping strategies to enhance both performance and well-being**.**

## 1 Introduction

Individuals in social settings and workgroups are highly interested in how others perceive them, and they dedicate significant time and effort to shaping other's perceptions through their self-view [[1], [2], [3], [4]]. Social identity processes significantly influence employee behavior, a topic that has been studied in the literature but requires further exploration [[5], [6], [7]].

Individuals are encouraged to be mindful of how others view them to create, control, and sustain favorable impressions with their colleagues, as suggested in practice and management literature [8,9]. This will help them to manage relationships more effectively, which can lead to improved attitudes, performance [5,10,11], and compliance with their organization [12,13]. In organizations, individuals may experience discomfort when they believe their colleagues have a different perception of them compared to how they view themselves, [14]. This discrepancy might affect their social behavior [15]. Researchers described this concern as internal identity asymmetry [6] the belief that one's identity is mistaken. It is important to analyze identity asymmetry from an individual's viewpoint since our perceptions of how others perceive us greatly impact our behaviors and relationships [[16], [17], [18]].

According to identity theory, internal identity asymmetry is defined as the degree to which an individual perceives misidentification by their colleagues at work. It extends on self-discrepancy theory [15,19] by exploring the anxiety between an individual's self-perceptions and external perception and how others perceive their identity, which results in adverse effects and emotional consequences [20,21]. Literature revealed that an individual's perceptions of phenomena influence their response [22,23]. The misalignment perception highlights the necessity to re-negotiate either with oneself or with others, to reduce discrepancies and establish congruence [3,24,25].

In addition, self-verification theorists focus on cognitive and behavioral approaches to address asymmetry, but they pay less attention to the circumstances necessary for maintaining this asymmetry. As individuals experience various kinds of asymmetries [7,26], how they decide to face or avoid asymmetry has yet to be discovered with the help of additional factors. Hence this study incorporated cognitive theories of stress and coping [27] to determine specific proximal coping responses that an individual is inclined to utilize.

Previous research examined and discussed identity asymmetry with several aspects such as gender identity and leadership identity [24,26,28], and creative identity [29]. Moreover, employee behavior attributes [30], interpersonal relationship and well-being [7], identity conflict [31,32], customers and professional relationship-image discrepancies [33], identity threat & identity challenge [6] and workgroup conflict [23] has also been discussed. However internal identity asymmetry with its consequences during personal and professional-based transition has rarely been accounted. Therefore, this study aims to investigate the internal identity asymmetry on employee's work performance and well-being.

Individuals need to be aware of how their own and others' perceptions of them can affect their impact and behavior in the workplace [1,6]. It is critical to recognize that employees' self-perception may not align with how others perceive them [34,35]. However, this incongruence may not be significant if employees do not perceive or value this difference. The existing literature needs a thorough examination of the process of incongruence to shed light on its potential benefits and the circumstances under which it may be advantageous [5]. The present research will add new insight to the existing literature by examining the mechanism through which identity asymmetry impacts behavior and emotional outcomes. Internal identity asymmetries experience may change over time; some factors like tenure and authority may affect the experience of asymmetry, which can reduce the complexity of the experience. This allows employees to plan and organize for the possible tense situation of being examined and unwanted identities assigned.

A prior study [26] examined the experience of internal identity asymmetry for women leaders through qualitative interviews in the western context. However, the consequences of internal identity asymmetry for both male and female employees have yet to be discussed [35], how and when it may affect their behavior & emotional outcomes in multiple regions of Pakistan. Hence, there is an urgent need to conduct scholarly research beyond the western context to capture the contextual difference in the eastern context, i.e., Asia.

The context for this study (Pakistan) is characterized as a collectivist country according to Hofstede's Cultural Survey [36]. Pakistan has a power distance score of 55, indicating a collectivistic society (score 5) where individuals with less authority typically defer to top management. Pakistan scores 70 on the uncertainty avoidance dimension, reflecting a culture that prefers to avoid uncertainty, leading to a constant state of stress and fear of the future. Furthermore, Pakistan is classified as a restrained society with a score of zero, which can be attributed to economic conditions and high-risk circumstances, resulting in strict control of desires [36]. Hence, people in Pakistan give more importance to relationships, and more focus on family than work; high uncertainty avoidance culture tend to take less risk [37], have low self-efficacy, and have moderate acceptance of power distance [38]. In addition, interdependent jobs, fewer employment opportunities, and the desire for secured jobs encourage significantly greater tolerance for mistreatment [39,40]. Therefore, it's curious to investigate the impact and mechanism of identity asymmetry on employee behaviors and feelings in the workplace of Pakistan.

The present research has several benefits, being a timely and relevant topic for both theory and practice. First, a recent study [26] has discussed the experience of internal identity asymmetry from a gender perspective, i.e., for women leaders in the western context. There is a need to discuss the experience of asymmetries in Asian context. This research, however, does not only apply to females; everyone should be encouraged to consider how they view, categorize, or refer to their colleagues in organizations.

Next, this research will also be contributed to introduce the physiological distress variable, which emerges as a consequence [29] during the internal identity asymmetry process. This is also a novel contribution, and there have yet to be any studies on the experience of internal identity asymmetry. We will also unpack the various coping strategies employed in response to this misidentification to avoid or react deliberately to the asymmetry experience. It will also contribute to when employees need to adapt these coping strategies and how they will cope with psychological distress caused by the experience of asymmetries. This will highlight the importance of coping strategies in improving the performance and well-being of employees at the workplace of multiple regions of Pakistan.

In addition, there is a lack of empirical studies to explore the impact of internal identity asymmetry on their outcomes [7,24,26,41,42]. Hence, this study will explore the causal relationship among internal identity asymmetry with behavior and emotional outcome, i.e., performance and well-being at the workplace, by explanatory research technique. This research would allow us to look at trends and differences in their careers as well as various industries and regions in Pakistan.

This study will explore the causal relationship between an individual experience of internal identity asymmetry on performance and well-being in the workplace of Pakistan. In addition, the study will also focus on examining how they will cope with psychological distress, caused by the experience of asymmetries. The researcher will also investigate the moderating effect of coping strategy on performance and well-being. In sum, the researcher seeks to examine the direct, indirect & moderating effects of internal identity asymmetries on their positive and negative outcomes by answering the following specific questions;

1. Does Psychological Distress mediate the effect of Internal Identity asymmetries on employee Performance & Well-being?
2. Does Coping Strategies moderate the effect of Psychological Distress with the outcomes of Internal Identity asymmetry i.e., Performance & Well-being?

The subsequent sections of this work are structured in the following manner: In the following part, a review of the existing literature is conducted, accompanied by a theoretical foundation for the suggested framework. Subsequently, a detailed account of the method is provided, followed by an analysis of the results, and an in-depth discussion of the findings and contributions.

## 2 Literature review and hypothesis development

This study is grounded on identity and self-verification theory, wherein internal identity asymmetry is described as the phenomenon of misidentification in the workplace. Individuals perceive that other incorrectly or unfavorably identify them, while ignoring identities that are potentially significant to them. Furthermore, it incorporates cognitive theories of stress and coping model to ascertain specific proximal coping responses that an individual is inclined to employ.

### 2.1 Internal identity asymmetries and psychological distress

When individuals perceive that others hold an incorrect perception of their work-related identity, they experience internal cognitive dissonance, which is the discomfort caused by contradictory cognitions [43,44]. They experience this cognitive dissonance as they navigate divergence between their own perception of their work-related identity and how others perceive it [7,45]. Consequently, Individuals often assume that their coworkers unintentionally recognize their work-related identity. As individuals strive to resolve internal inconsistencies, dissonance is associated with increased psychological stress, tension, and decreased well-being [46]. However, Asymmetry represents an inconvenient or distressing experience, yet the evaluation of asymmetry may vary as some perceive it as highly negative and others as positive [47] which further influences the potential response and outcome of individuals [7].

According to an in-depth qualitative analysis by Meister et al. (2017) [26], Internal identity asymmetry is assessed through two primary constructs: personal-based asymmetry and professional-based asymmetry. Personal-based asymmetries pertain to an individual's personal attributes, such as competence, gender, or character. For example, many women encountered personal based asymmetry linked to their gender role, particularly in organizations where they were minorities [26]. Gender identity asymmetry frequently correlated with high level of personal based asymmetries. Many individuals perceived that highlighting gender led others to view them as incompetent or incapable of fulfilling their roles. On the other hand, professional-based asymmetries encompass discrepancies that do not directly relate to the individual's personal characteristics in the workplace. These discrepancies may involve how others perceive their work, team, department, organization, or profession. Individuals frequently associate themselves with their organization or profession, which may lead to low and high professional base asymmetries. This underscores the significance of perceptual asymmetries regarding one's profession, department, or team, as they can significantly impact individuals.

Psychological stress increases when the demands of the situation exceed the individual's perceived capacity to handle them, according to the stress and coping model proposed by Lazarus and Folkman (1984) [48]. In other words, the model emphasizes fit between a person & environment, and efficient coping is linked to the quality of fit between environmental requirements and the resources available to the person. Therefore, according to the above theoretical background, an employee will have psychological distress if they cannot handle the internal identity asymmetries (personal & professional based) at the workplace based on personal and social resources available to the employee. Consequently, the following hypothesis is developed;

H1-a There is a positive relationship between personal based asymmetry and psychological distress.

H1-b There is a positive relationship between professional based asymmetry and psychological distress.

### 2.2 Psychological distress and individual work performance & well-being

Previous research has shown that incongruent experiences have negative consequences, especially when it comes to an individual's identity. These incongruent experiences have been linked to detrimental effects leading to more stress, anxiety, and a decrease in the health & well-being of individuals [49,50]. In terms of self-image, discrepancies among an individual's expected and perceived external image have been linked to reduced psychological health and strained interpersonal relationships [33,51]. Furthermore, research on interpersonal congruence highlights the positive outcomes of increased workplace congruence, including creativity, turnover, performance, satisfaction, and the quality of interpersonal relationships [[52], [53], [54]]. Internal identity asymmetry is a discomfort experience or tension when employees perceive a discrepancy between their own view of their work-related identity and how it is inaccurately perceived by others, leading to potential distress [50]. According to the literature, asymmetries may be intentionally or unintentionally created [7], and no matter its accurate or not, they have to cope with negative experiences. Accordingly, the researcher argues that feeling misidentified influences the individual's identity, which may lead to negative experiences, and employee performance and well-being may be affected if it remains unsolved.

When assessing the overall performance of an organization, the performance of a particular individual is considered critical, therefore companies are keen to find dedicated and committed employees who can carry out and achieve responsibilities as needed. Aguinis and Kraiger (2009) [55] describe individual work performance as an individual's capacity to fulfill their job responsibilities through utilizing their expertise, knowledge, attitude, and motivation. With this, Individual work performance is associated with the behaviors and actions of employees rather than solely focusing on the outcomes of those actions [56,57].

Prior studies have highlighted a negative connection between perceived stress and job performance [58]. Wang et al. (2023) [29] suggested that through psychological strain, creative performance may diminish as a result of creative identity asymmetry. Therefore, psychological distress may reduce the capability of employee skills, but here we will examine their negative impact on performance and well-being. In this case, when there is more psychological distress, there would be more hazardous behavior, which will harm the performance and well-being of employees. This leads to the development of the following hypothesis.

H2 There is a negative relationship between psychological distress and (a) individual work performance (IWP) & (b) well-being (WB).

### 2.3 Mediating effect of psychological distress

Internal identity asymmetry is an unavoidable tension within organizational workgroups and evaluated as a challenge and threat that can lead to both outcomes, i.e., constructive or destructive. Thus, it is crucial for employees to understand these interpersonal identity processes to achieve positive outcomes for individuals, groups, and organizations [6]. Intra-individual tensions, according to self-discrepancy theory [15], may result in negative affect and emotions such as punishment, rejection, agitation-related emotions, fear, and a sense of threat or presentment. Prior research Wang et al. (2023) [29] investigated the mediating role of psychological strain between creative identity asymmetry and creative performance. This study explores how an increasing misalignment between an individual's perceived creative role and their actual creative performance leads to decreased creative performance. This connection was found to be influenced by the presence of psychological strain.

In the above, it has been argued that Internal identity asymmetries have a negative impact on psychological distress (H1-a, b) as individuals are classified inappropriately by others. The researcher also hypothesized that psychological distress (H2-a, b) will have a negative impact on performance and well-being. The chain of relationships clearly shows that the extent of internal identity asymmetries, i.e., personal and professional, can positively impact an individual's work performance & Well-being. Hence, more asymmetries will impact subjective performance and well-being through psychological distress. Therefore, the researcher proposes that;

H3-a Psychological distress mediates the relationship between personal based asymmetry and an individual's pork performance (IWP).

H3-b Psychological distress mediates the relationship between personal based asymmetry and individual's well-being (WB).

H3-c Psychological Distress mediates the relationship between professional based asymmetry and individual's work performance (IWP).

H3-d *Psychological Distress mediates the relationship between 0rofessional based symmetry and well-being (WB*).

### 2.4 Moderating effect of coping strategies and performance & well-being

Self-verification theorists focus on cognitive and behavioral strategies to address asymmetry, but they overlook situations where an individual may choose to maintain an asymmetry. We can understand better how people cope with internal identity asymmetry by integrating cognitive theories of stress and coping [27,48,59]. Individuals cope with external and internal stressors, including internal identity asymmetry [48] prompting individuals to utilize various cognitive and behavioral responses [44,60,61]. While individuals generally desire congruence [60], they may maintain an asymmetry relatively to resolve it [7]. Therefore, it has been argued that psychological distress can have a negative impact on coping strategies resulting from verifying their salient work-related identities.

Individuals engage in a two-component cognitive process known as primary and secondary appraisal [48,59,62] while dealing with a discrepancy in one's sense of self (internal identity asymmetry). Primary appraisal evaluates the significance of the asymmetry to their well-being and future goals whereas how to deal or handle with the asymmetry can be determined in secondary evaluation. Whether the asymmetry is perceived as a threat (harm or loss) or challenge (benefits or gain) is determined by these simultaneous appraisal processes [27]. Individuals then engage in either maintenance or resolution coping responses due to this appraisal. Intentional or not, the presence of asymmetry can act as a source of stress, causing them to engage in cognitive assessment processes. Despite their intentions, asymmetry itself can be a stressor, prompting individuals to undergo cognitive assessment processes. This shows that psychological distress will positively impact performance and well-being and intersection with coping strategies to reduce the distress [63]. Meister et al. (2023) [42] research revealed how assessment processes on individual attitudes and performance outcome's moderate internal identity asymmetry. Their study demonstrated that negative affect and coping resource appraisals combined to influence the relationship between internal identity asymmetry and actual performance outcomes. The above-mentioned theoretical background contributes to the development of the following hypothesis;

H4 Coping strategies moderate the relationship between psychological distress and individual outcomes i.e. (a) individual's work performance (IWP) & (b) well-being (WB).

## 3 Conceptual framework

In this study, internal identity asymmetries are divided into main salient types of asymmetries w.r.t to personal and professional base asymmetries. *Personal based asymmetries* related to personal identities such as skills recognition, gender, personality & communication. Professional based asymmetries are the discrepancies in how individuals perceive others to see their role, profession, department, or organization [26].

According to the model of stress and coping [48,64], *Psychological stress* arises if the demands of the situation go beyond the perceived capacity of an individual to deal with it. In other words, the model emphasizes fit between a person & environment, and efficient coping is linked to the quality of fit between environmental requirements and the resources available to the person. Therefore, according to the above theoretical background, an employee will have psychological distress if they are unable to handle the internal identity asymmetries (personal & professional) at the workplace based on personal and social resources available to the employee.

**Fig. 1:**
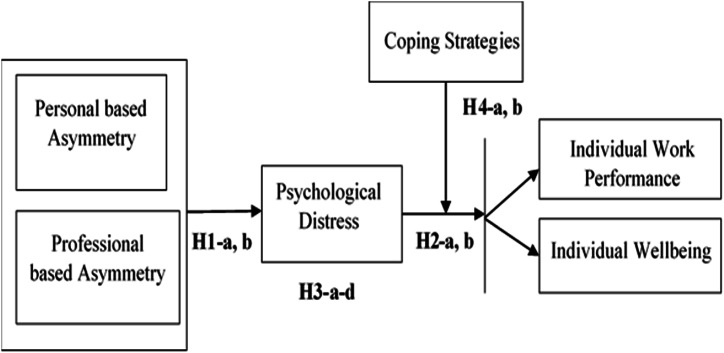
Conceptual framework.

In this study, the researcher focuses on individual behavior and emotional outcomes i.e., Performance and well-being of employees. *Individual work performance* is described as an individual's ability to fulfill their work duties through required expertise, knowledge, attitude, and motivation. Individual work performance is characterized as employee behaviors or actions rather than the results of these actions [56,65]. *Individual Well-being (WB)* can be regarded as work-related happiness, experiencing positive emotions at work [66] such as joy, excitement, enthusiasm, and contentment [67]. The diagram illustrated in Fig. 1 provides a detailed visual representation of the conceptual model.

## 4 Research methodology

### 4.1 Research design

A cross-sectional survey was performed to explore the relationship between the variables. The sample comprises individuals who are 18 years old and above, compared to those chosen using a non-probability convenient sampling method. The data were gathered by the use of a standardized questionnaire, which included demographic information and evaluation tools for internal identity asymmetry. The study used statistical analytic techniques, namely partial least squares-based structural equation modeling (PLS-SEM), to investigate the relationships and factors that influence internal identity asymmetry. The details of research methodology are outlined in the following section.

### 4.2 Participants and procedure

This survey was performed in Pakistan where employees from different organizations (i.e., Manufacturing, academia, health care, IT, Media, hospitality, Non-Profit & many more) have been considered as the study population. Because of its diverse population in terms of ethnicity, language, religion, and geography, Pakistan offers an excellent setting for studying internal identity asymmetry and its effects on social cohesion, nation-building, and governance. However, we obtained samples from populations applying a non-probability convenient sampling technique. This technique was chosen because it is cost-effective and time saving. In addition, respondents were obtained based on the positions of three levels of management i.e., front, middle, and top level of management. The statistical formula (Formula-1) below was used to determine the sample size. It is assumed that the sample would be 95 % likely to yield an estimate at a certain specific point. Precision is defined in the estimate as the tolerated noise margins.

(1) $$n=\frac{z^{2}\;p\left(1-P\right)}{d^{2}}$$

Where n is the sample size; *z* is the two-sided normal variate; *p* is the indicator percentage and d are precision.

The minimum required sample size is 384, with 5 % precision, 95 % confidence level, and the most conservative estimate of predictor percentage (50 %). However, a total of 393 valid responses were used for further analysis.

### 4.3 Procedure for data collection

The research employed the survey-based technique for collecting primary data. The questionnaire method was chosen for several reasons, first, this is an explanatory study illustrating basic quantitative research techniques. Secondly, the Questionnaire approach in the explanatory analysis examines the cause-effect relationship [68]. Hence, the questionnaire is more compatible as the data collection tool over interviews and observation. Moreover, an investigative protocol helps ensure anonymity and uncover the facts without evoking fear.

Primary data were collected by using a close-ended questionnaire to measure the causal relationship between employee's experiences of internal identity asymmetry and their consequences on performance and subjective well-being in the workplace of Pakistan. The questionnaire consisted of two sections. Participant's demographic information i.e., gender, age, educational background, occupation, years of experience, sector, and industry were listed in section one. The second section discussed data collection using a five-point scale on the construct under analysis. The survey was administered in English and all measurement items were developed in English. Given that English serves as the official language for communications in all business settings inside Pakistan, there is no need to translate into the local language. Prior studies in Pakistan have also used English questionnaires [37,69]. The survey was approved by the ethical committee of the School of Management, Northwestern Polytechnical University, Xi'an, China (Ref#: SOM-72-07-2022).

Data were collected through a web-based survey that reached employees from various industries across Pakistan. The existing body of literature indicates that data collected from different industries may provide a reasonable level of generalizability to study outcomes [[70], [71], [72]]. The data collection process included contacting the sample group by email. The researcher sent an Email to the relevant HR or Department manager, with the hyperlink to the online survey. The demographic composition of the study participants is given in Table 1.

**Table 1:** Demographic analysis.

Socio-Demographic	Response	Frequency	Percentage (%)
Gender	Male	270	69
	Female	123	31
Age (years)	20–25	58	14.8
	26–30	124	31.6
	31–35	97	24.7
	36–40	52	13.2
	41–45	36	9.2
	46–50	14	3.6
	≥51	12	3.1
Marital status	Unmarried	155	39.4
	Married	235	60
	Divorced	3	0.8
Education	High School	7	1
	Bachelors	89	23
	Master	220	56
	Doctorate	77	20
Experience (years)	1–3	127	32.3
	4–6	66	16.8
	7–9	51	13.0
	10–12	45	11.5
	13–15	39	9.9
	≥16	65	16.5
Occupation	Front Level Officer	86	21.9
	Supervisor/Lecturer	107	27.2
	Assistant Manager/Executive	67	17.0
	Manager/Sr. Executive	91	23.2
	GM/Country Manager	19	4.8
	Dy. Director/Professor	11	2.8
	COO/CFO/Dean	7	1.8
	CEO/Managing Director	5	1.3
Employment status	Contractual	186	47.3
	Permanent	207	52.7
Sector	Public	121	30.8
	Semi Govt.	65	16.5
	Private	152	38.7
	Corporate	23	5.9
	Multinational	32	8.1
Industry	Academia &Science	112	28.5
	Financial Services	52	13.2
	Health Care	48	12.2
	Manufacturing	39	10
	Technology	70	18
	Telecommunications & Media	24	6
	Energy	10	2.6
	Hospitality	28	7
	Non-Profit	10	2.5

Table 1 illustrates a consisted reflection of the study. In this study, 69 % participation of males and 31 % of females, almost 46 % of respondents were less than 30 years old 38 % belonged to the age group 31–40, 13 % were from 41 to 50, and only 3 % from above 50 age group. About 60 % of respondents are married, 39 % are unmarried and almost 1 belong to another category i.e., divorced. In the context of education, 56 % of respondents have a master's degree, 23 % have an undergraduate and 19 % have a Ph.D. Degree. Table 1 represents the highest number of respondents having experience of 1–6 years (49 %), 7–12 years (24.5 %) and 26.5 % of respondents have more than 13 years. We have categorized 3 levels of management i.e., front level officer supervisor, lecturer, assist. manager & executive into the front level, middle-level management into all types of managers, sr. executives, professors, Dy. director and COO, CFO/Dean, CEO & MD categorized into top-level management. According to this categorization, 66 % of respondents are from the front level, 40 % are from the Middle level and 4 % are from Top level management. About 28.5 % of the respondents belong to academia & science, 18 % belong to technology, following financial services (13 %), health care (12 %), and manufacturing (10 %). Few respondents from hospitality (7 %) telecommunications & media (6 %), energy (2.5 %), and non-profit (2.5 %) industries were also part of the study. Respondents belong to different sectors of Pakistan i.e., the public sector (31 %), private sector (39 %), semi Govt (16 %), corporate (6 %), and 8 % from multinational organizations.

### 4.4 Procedure of data analysis

This study applied partial least square-based structural equation modeling [73] using the Smart PLS software package to validate the proposed research model and develop hypotheses and SPSS 23.0 software also has been used. PLS-SEM is a flexible and successful multivariate analytical research tool that can be used to describe and analyze underlying structures and their relationships using participant observation results. PLS-SEM is strongly associated with multiple regression [74]. The basic research model can be validated and analyzed using this method with as few as 100 observations as samples. This study aims to examine the theoretical model by investigating the causal relationship between internal identity asymmetries and their outcomes with the mediating role of psychological distress.

### 4.5 Instrument measures

Due to the novelty of the research context in the social sciences, there was a demand for more established instruments. This study used contextualization based on established data from prior literature, resulting in modifications to most of the relevant sub-constructs and instruments. A summary of measures of constructs, items, and a short explanation with references is given in Appendix A.

Internal identity asymmetry is measured by two major constructs i.e., personal based asymmetry and professional based asymmetry [26]. There is no specific measurement scale for assessing personal/professional based asymmetry. Conceptualization of these constructs emerged from pre-existing instruments [[75], [76], [77]] and is adapted to measure each construct. The researcher slightly modified the original version and re-evaluated it accordingly. Discrimination scale is intended to measure more chronic, routine, and relatively small unfair treatment experiences [78]. Therefore, we adapted specific dimensions for personal and professional base asymmetry and slightly modified them. Participants were asked to report how they get misidentified based on gender, personality, KSA's, and department related factors like promotion, pay raise & evaluation. Each item was comprised on a five-point Likert scale, with 5 being strongly disagree and 1 being strongly agree.

*Personal Based Asymmetry* (PBA) consists of 6 items such as gender [75], personality [77], skills & abilities [75] and harassment [76]. Cronbach's alpha coefficient of all 6 items in the present study was 0.791.

*Professional Based Asymmetry* (Pr. BA) consists of 6 items including department & team, promotion & pay raise, and evaluation adopted from the Chronic Work Discrimination and Harassment scale [76]. Cronbach's alpha coefficient of all 6 items in the present study was 0.865.

*Psychological Distress***:** A simple measure of psychological distress is the Kessler Psychological Distress Scale (K6) [79] to analyze the person's emotional state this scale employs 6 questions. Each item is scored from 0 to 4 where 0 = None of the time and 4 = All of the time. The Japanese version's reliability and validity had previously been confirmed [80]. Cronbach's alpha coefficient was reported as 0.813 in this study.

*Coping Strategies***:** A brief COPE scale which is the shortened version of the COPE inventory was used to assess the coping strategies. To assess individuals' strategies for coping with psychological stress, we applied 9 scales of the Brief COPE Inventory designed by Carver (1997) [81]. problem-focused vs emotion-focused coping skills can be analyzed through a brief COPE Scale. 14 items for problem-focused strategies were taken such as active coping, use of emotional support, use of instrumental support, positive reframing, planning, and acceptance. 5 items for emotion-focused strategies such as included self-distraction, denial, and behavioral disengagement. Participants responded on a 5-point Likert scale, ranging from 1 = I haven't been doing this at all to 5 = I've been doing this a lot). In the present study, Cronbach's alpha coefficient was reported as 0.863.

*Individual Work Performance***:** A scale of Individual Work Performance (IWP) 0.3 [56,57] was employed to measure the work performance of individuals. In total, 10 items to test individual work performance were obtained through the scale chosen. The IWP rating scales used a 5-point scale, with 5 representing seldom, sometimes, frequently, often, and 1 representing always. Cronbach's alpha coefficient of IWP was 0.86.

*Individual Well-being***:** Individual well-being is measured by 7-item scale of happiness developed by the Work-Related Affective Feelings Scale (WORAF) [82] and Job-Related Affective Well-Being Scale (JAWS) [83]. Emotions like enthusiasm, joy, fulfillment, and excitement may relate to happiness [67], Work-related happiness, experiencing positive emotions at work [66], may be considered as job satisfaction. Feelings of job satisfaction and fulfillment should lead to a higher level of happiness in an employee. According to Jaworek et al. (2020) [82], an employee who expresses a high level of happiness at work has demonstrated a sense of job satisfaction and accomplishment. We employ a 5-point Likert scale, with 5 denoting extremely often and 1 denoting never and Cronbach's alpha coefficient was reported as 0.877.

## 5 Result & analysis

### 5.1 Normality test

This study used the SPSS 23.0 package to check the univariate normality of each variable using the skewness and kurtosis approach [84]. The measurements of skewness and kurtosis, along with their corresponding standard errors, are shown in Table 2;

**Table 2:** Skewness and kurtosis statistics.

	N	Std. Deviation	Skewness		Kurtosis	
	Statistic	Statistic	Statistic	Std. Error	Statistic	Std. Error
**Personal based Asymmetry**	393	0.74368	0.375	0.123	−0.080	0.246
**Professional based Asymmetry**	393	0.82826	0.475	0.123	0.036	0.246
**Psychological Distress**	393	0.72396	−0.418	0.123	0.326	0.246
**Coping strategies**	393	0.64235	0.638	0.123	1.200	0.246
**Wellbeing**	393	0.86727	0.691	0.123	0.428	0.246
**IWP**	393	0.78768	0.725	0.123	0.326	0.246

Note. IWP − Individual Work Performance.

Table 2 shows the absolute skewness value of personal asymmetry, professional based asymmetry, psychological distress, coping strategy, well-being, and individual work performance (IWP) are within +1 and −1. Therefore, these variables' data are sufficiently normally distributed [85]. Though the coping strategies' kurtosis was slightly above 1, even when the skewness value is less than 2 and the kurtosis value is less than seven the assumption of normality can be met [86].

### 5.2 Control of common method bias (CMB)

The data of the present study were self-reported questionnaires. Hence there is potential for common method bias (CMB) [87]. This concern becomes strongest when perception measures (both dependent and focal informative factors) are taken from the same respondent, this can lead to false internal consistency that refers to the association between variables created by their shared origin [88].In order to calculate the multicollinearity, variance inflation factors (VIFs) were calculated to identify the problem of common method bias (CMB). The Collinearity test is therefore used to identify the bias in the common method that leads to variance inflation factors (VIFs). When the maximum collinearity measure is ≤ 3.3, the model can be assumed to be free of common method bias while when the values of VIFs are >3.3, then the model is corrupted by common method bias [89]. According to findings from Appendix B, all VIF values are below the cut-off value of 3.3. A VIF value less than 3.3 is a good indicator, therefore the model is free of common method bias (CMB). It can therefore be assumed that common method bias did not influence participants' responses.

### 5.3 Kaiser-Meyer-Olkin (KMO) test

Most of the scales used in this study were developed in Western nations so the measurement scale and their validity should be tested and checked. Therefore, the important statistics for calculating sampling adequacy are Kaiser-Meyer-Olkin (KMO). This index compares the magnitudes of the coefficients observed for the correlation to those of partial correlation coefficients. Small values (less than 0.5) in KMO indicate that other variables cannot explain correlations between pairs of variables and may not be appropriate in factor analysis. KMO test will be performed on each construct, for sample Adequacy in below Table 3. According to Table 3, none of the assumptions were violated, and all six variables' KMO values are greater than the 0.60 minimum requirements. In terms of Bartlett's test of sphericity, the findings in Table 4 suggest that scores for all variables were statistically significant (p = 0.000). Therefore, factor analysis was acceptable for the data collected in this study.

**Table 3:** KMO and Bartlett's Test for each Construct.

EFA's Assumptions	PBA	Pr. BA	PD	CS	IWP	WB
KMO	0.784	0.832	0.833	0.800	0.848	0.887
Bartlett's Test	0.000	0.000	0.000	0.000	0.000	0.000

**Table 4:** KMO and Bartlett's test.

KMO and Bartlett's Test		
Kaiser-Meyer-Olkin Measure of Sampling Adequacy		0.899
Bartlett's Test of Sphericity	Approx. Chi-Square	11588.458
	df	1596
	Sig.	0.000

### 5.4 Measurement model

In this study, content validity, convergent validity, and discriminant validity were used to assess the measurement model [90]. Factor loading, Cronbach's alpha, composite reliability (CR), average variance extracted [69], and discriminant validity using both the Fornell-Larcker and Heterotrait-Monotrait (HTMT) ratio criterion [91] were used in this study.

The goodness of fit parameter i.e. the standardized root mean residuals (SRMR), has been used to measure the general fitness of the PLS-SEM measurement model that Hasan et al. (2019) [92] have recommended. A recent simulation study shows that completely defined models will yield 0.06 and higher SRMR values [93]. Thus, values up to 0.08 are considered acceptable for PLS path analysis. The SRMR value in this study is 0.065, which is less than the threshold level of 0.08, which lies in the high chance of expurgatory power as proposed by Henseler et al. (2014b) [94]. Thus, the model fit is suitable for analysis as the indicators were satisfactory.

According to Hair Jr et al. (2014) [95], content validity is a test of the contingency between the items' measured characteristics and the constructs that were evaluated by the panel of experts. By examining composite reliability (CR) and average variance extracted [90,95] and Cronbach's alpha [96], the convergent validity is assessed in the second step. Nevertheless, Cronbach's alpha values are regarded as essential for checking the internal consistency of the items. According to Table 5, the result shows that all the values of Cronbach's alpha are greater than 0.70 suggesting a satisfactory level [97]. Similarly, composite reliability (CR) observed in Table 5 that all constructs have values above 0.7 is also accepted as suggested by Baozzi and Yi (1988) [98]. Convergent validity is confirmed [99] when all the outer loading values are greater than 0.70, and the average variance extracted is above 0.50. Appendix B indicates that several items show significant factor loading concerning the converging validity, although some items in the testing instrument did not meet the threshold of 0.70 and were therefore excluded. Thus, there is no obligation that outer loading estimates must be higher than 0.7. Consistently, all item ranges had standardized factor loadings of 0.7 or higher, as Chin et al. (1997) [100] and Hair et al. (2006) [101] recommended.

**Table 5:** Convergent validity analysis.

Factor	Cronbach's Alpha	Rho_A	CR	AVE
Personal Based Asymmetry	0.761	0.762	0.848	0.583
Professional Based Asymmetry	0.865	0.867	0.899	0.599
Psychological Distress	0.804	0.815	0.872	0.631
Coping Strategy	0.863	0.875	0.897	0.594
Individual work Performance	0.86	0.864	0.896	0.589
Wellbeing	0.894	0.895	0.919	0.656

Note. CR- Composite Reliability, AVE-Average Variance Extracted.

Further, the Fornell–Larcker criterion and the examination of cross-loadings are the key approaches to determine discriminatory validity by using the square root of AVE based upon the recommendation of Fornell and Larcker [102]. According to the below Table 6 the values of discriminant validity were found to be satisfactory and all constructs had higher square roots than their correspondence values to the other constructs. Fornell-Larcker criterion and the examination of cross-loadings approaches do not consistently detect the discriminant validity in typical research situations and it also requires measuring the Heterotrait–Monotraiti (HTMT) ratio [92,94]. In conclusion, The HTMT criteria were then determined for each pair of the construct. The HTMT results are shown in Table 6, where the values suggested that there were issues with discriminant validity in Professional based asymmetry to the HTMT_0.85_ criterion [92,94]. However, when considering HTMT0.9, these factors-maintained discriminant validities.

**Table 6:** Discriminant validity analysis.

	Fornell-Larcker Criterion						Heterotrait-Monotrait (HTMT) ratio					
	CS	IWP	PBA	Pr.BA	PD	WB	CS	IWP	PBA	Pr.BA	PD	WB
CS	**0.771**											
IWP	0.415	**0.768**					0.470					
PBA	0.062	0.306	**0.763**				0.120	0.375				
Pr.BA	0.059	−0.237	−0.507	**0.794**			0.155	0.277	0.639			
PD	0.096	0.305	0.754	−0.51	**0.774**		0.133	0.352	0.881	0.601		
WB	0.225	0.476	0.500	−0.483	0.494	**0.810**	0.247	0.542	0.605	0.570	0.564	

Note. PBA − Personal based Asymmetry, Pr.BA − Professional based Asymmetry, PD − Psychological Distress, CS − Coping Strategies, IWP − Individual Work Performance, WB − Well-being.

### 5.5 Structural model and path analysis

The structural model was performed after evaluation of the outer model which is a prerequisite for this analysis, due to the appropriate level of validity. According to Hair Jr et al. (2016) [103], an assessment of the inner model direct relationship was performed via the value of standard error, path coefficient β value, p-values, and t-values to decide significance and insignificant. Bootstrapping procedure in Smart PLS 4.0.9.8 software was used to test the developed hypothesis (direct, mediating, and moderating effect) and research model (Fig. 1). Table 8 shows the total and indirect effects of construct PBA, Pr.BA, PD, IWP & WB. We found in path analysis (Table 8) that there is a significant negative relationship between personal based asymmetry (β value = −0.284, p < 0.001) and professional based asymmetry (β value = −0.296, p < 0.001) with psychological distress (H1-a, b). Moreover, psychological distress (H2-a, b) has a significant negative relationship with both outcomes i.e., individual's work performance (β value = −0.275, p < 0.001) and well-being (β value = −0.491, p = p < 0.001). Thus, Hypothesis H1-a, b & H2-a, b is proved with significant value p < 0.001.

**Fig. 2:**
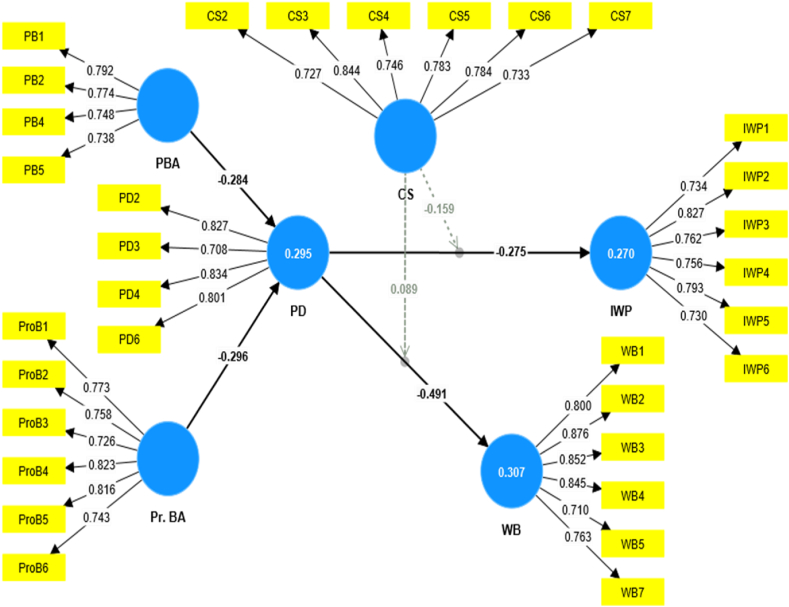
Result of path analysis.

PLS-SEM procedures also yield an R-square for evaluating the variance of the model's configuration. Therefore, R-square values will determine the explanatory power of the model (Table 7). According to Sarstedt et al. (2014) [104], the R^2^ values of 0.75 (substantial), 0.50 (moderate), and 0.25 (weak) reflect relationship respectively. The interpretation of the value of R^2^ in PLS-SEM is the same. Therefore, three dependent variables have significant variances i.e. psychological distress (R^2^ > 0.295), individual performance (R^2^ > 0.270) & well-being (R^2^ > 0.307). The PLS analysis & path coefficients are presented in more detail in Fig. 2.

**Table 7:** Predictive relevance analysis of Psychological Distress (PD).

Construct	R square	Q Square
**Individual Work Performance (IWP)**	0.270	0.150
**Psychological Distress (PD)**	0.295	0.180
**Wellbeing (WB)**	0.307	0.196

**Table 8:** Results of the path analysis of Psychological Distress (PD).

Path	*β*	T-Statistics	*P*-values	SE	Bias-corrected CI	
					2.5 %	97.5 %
**PBA →PD**	−0.284***	4.332	0.000	0.003	−0.409	−0.156
**Pr. BA →PD**	−0.296***	4.415	0.000	0.003	−0.420	−0.156
**PD →IWP**	−0.275***	6.104	0.000	0.002	−0.359	−0.182
**PD→ WB**	−0.491***	10.198	0.000	0.002	−0.582	−0.392
**PBA →PD → IWP**	0.078***	3.415	0.001	0.001	0.038	0.127
**PBA → PD→ WB**	0.139***	3.608	0.000	0.002	0.070	0.219
**Pr.BA → PD → IWP**	0.082***	3.545	0.000	0.001	0.041	0.131
**Pr. BA → PD-→ WB**	0.145***	4.037	0.000	0.002	0.077	0.219

Note. PBA − Personal based Asymmetry, Pr.BA − Professional based Asymmetry, PD − Psychological Distress, CS − Coping Strategies, IWP − Individual Work Performance, WB − Wellbeing, SE-Standard Error.

Finally, a Q square (Q^2^) test was performed to determine the prediction relevance of the proposed model. The model has very high prediction relevance is very high when Q^2^ is greater than zero, indicating demonstrating outstanding [105]. IWP = 0.150, PD = 0.180 & WB = 0.196 in Table 7 indicate a good model fit and the required value for the Q^2^. Therefore, the model describes a moderate explanation of the overall research problem, and it is appropriate.

### 5.6 Mediating effect of psychological distress

To test the mediation effect relationship, the study adhered to the recommendations of [106,107]. According to Rahi et al. (2019) [107], the bias-corrected confidence interval (CI) is used to determine the lower and higher value that results from the indirect effect. When the bootstrap confidence interval (CI) is both above and below zero, bias-corrected estimates are utilized in the estimation process. The results of the bootstrap analysis of the mediating effect of psychological distress are presented in Table 8.

Personal based asymmetry (H3-a, b) has an indirect effect on individual outcomes i.e., individual work performance (β = 0.078, SE = 0.001, p < 00.001) and well-being (β = 0.139, SE = 0.002, p < 00.001) mediated by psychological distress. It does not imply zero due to the indirect effects of PBA →PD → IWP, bias-corrected boot CI (LL = 0.038, UL = 0.127) & PBA → PD→ WB (LL = 0.070, UL = 0.219) respectively. These findings suggest that psychological distress mediates the relationship between personal based asymmetry with both outcomes I.e., individual's work performance (IWP) and well-being (WB) in a positive and significant manner.

Professional based asymmetry (H3-c, d) has also an indirect effect on individual outcomes i.e., IWP (β = 0.082, SE = 0.001, p < 00.001) and well-being (β = 0.145, SE = 0.002 p < 00.001) mediated by psychological distress. Bias-corrected confidence interval (CI) of Pr.BA → PD → IWP (LL = 0.041, UL = 0.131) & Pr.BA → PD → WB (LL = 0.077, UL = 0.219) doesn't indicate zero, Therefore, psychological distress mediates the significant positive relationship between professional based asymmetry with both outcomes i.e., Individual's Work Performance & Well-being. Hence Hypothesis H3-a, H3-b, H3-c & H3-d are proved with significant value p < 0.001.

### 5.7 Moderating effect of coping strategy

We adhered to the prescribed guidelines [74,101] in order to investigate how coping strategies [42] affect the association between psychological distress (PD) and individual's work performance (IWP) and well-being (WB). According to the findings of the bootstrap analysis following Hair Jr et al. (2017) [74], the confidence intervals (CIs) did not encompass zero, indicating a significant or accepted moderation effect. When the moderation effect extends beyond the zero threshold, it is not considered acceptable. In this study, the moderation effect was assessed at a significance level of 0.05.

**Fig. 3:**
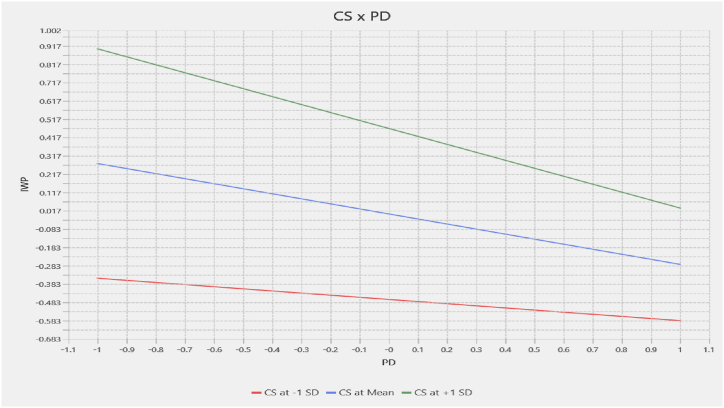
Coping strategies & Psychological Distress interaction effect on IWP.

Table 9 demonstrates the significant negative impact of the PD*CS interaction (β = −0.159; p < 0.005) with IWP. Whereas CS*PD interaction with well-being (β = 0.089, p = 0.109) was found insignificant in statistical terms so H4-b is rejected. Therefore, only H4-a is supported that coping strategies moderate the association between psychological distress and IWP. A visual inspection of Fig. 3 shows the moderation interaction graph, in order to foster an explicit understanding of the moderation, where green, blue, and red lines signify the moderator's high, mean, and low positions. Results in Fig. 3 indicate that coping strategies invert the negative relationship between psychological distress and Individual work performance. This also signifies that under a healthy workplace environment, when coping strategies are more effective, there is a positive effect on an individual's work performance. The size of the moderating effect (0.004) of coping strategies is medium, Therefore, H4-a is supported.

**Table 9:** Results of the path analysis of Moderating effect.

Path	*β*	T-Statistics	*P*-values	SE	Bias-corrected CI	
					2.5 %	97.5 %
CS*PD → IWP	−0.159***	3.443	0.004	0.002	−0.248	−0.066
CS*PD → WB	0.089	1.602	0.109	0.002	−0.020	0.201

Note. PD − Psychological Distress, CS − Coping Strategies, IWP − Individual Work Performance, WB − Well-being.

## 6 Discussion & conclusion

This study is conducted to examine the effect of internal identity asymmetries i.e., personal and professional based on their individual outcomes i.e., individual's work performance and well-being. Accordingly, research hypothesized the indirect effect of internal identity asymmetries on their outcomes through psychological distress and interacting effects of coping strategies on the relationship between psychological distress and individual's work performance & well-being.

In order to capture the indirect effect of internal identity asymmetries on their outcomes through psychological distress, four hypotheses were developed (H3-a to H3-d). Before this, we developed two hypotheses to capture the direct effect of personal & professional based asymmetries and psychological distress H1-a & H1-b. We also developed two hypotheses H2-a & H2-b to explore the direct effect between psychological distress and outcomes i.e., Individual Work Performance (IWP) & Subjective Well-being (WB). First, H1-a & H1-b refer to both personal & professional base asymmetries that will have an impact on psychological distress. Concerning this, the result revealed that personal based asymmetry (β value = −0.327, p < 0.01) and professional based asymmetry (β value = −0.273, p < 0.01) have a positive impact on psychological distress. These findings are consistent with the conceptual model of stress and coping [48] that an employee would experience psychological distress when his ability to cope is insufficient to deal with or manage the internal identity asymmetries (personal & professional based) at the workplace [7]. Second, H2-a & H2-b refer that psychological distress has a negative impact on both outcomes i.e., individual's work performance (β value = −0.285, p < 0.01) and well-being (β value = −0.500, p = p < 0.01). Individuals experience psychological distress when others incorrectly classify them, which can be caused by the negative evaluation of internal identity asymmetries, i.e. personal & professional [26]. Individuals may experience a range of negative emotions [108] as a result i.e., anger, frustration, and fear [49,109], as they believe they may lose something rather than gain anything.

Therefore, it impacts the employees' performance and well-being to some extent. Third, H3-a & H3-b refer that, psychological distress mediates the effect of personal & professional base asymmetries on an Individual's Work Performance (IWP) & Well-being. The result revealed that Personal based asymmetry has indirect effect on IWP (β = 0.093, p < 00.001), & Well-being (β = 0.163, p < 00.001) through psychological distress. Similarly, H3-c, & H3-d referred that Professional based asymmetry has indirect effect on IWP (β = 0.078, p < 00.001) & well-being (β = 0.136, p < 00.001) through psychological distress.

In this study, we found that psychological distress is created due to personal and professional experiences of asymmetries [26] so it may affect the performance and wellbeing of employees at the workplace. A recent study conducted by Wang et al. (2023) [29] examined how psychological strain mediates between creative identity asymmetry and creative performance. There has not been any other empirical research testing the effect of internal identity asymmetries i.e. personal & professional asymmetries on individual work performance and the subjective well-being of employees through psychological distress of employee. Thus, this is a novel finding in the existing literature and an expansion of a previous conceptual work of Meister et al. (2017) [26].

Results show that the interaction term of psychological distress and coping strategies on an individual's work performance is statistically significant (β = −0.139; p > 00.005). Therefore, the effect of psychological distress on an individual's work performance was stronger with coping strategies. This indicates how individuals can effectively manage stress and maintain or enhance their performance in the workplace. Whereas interaction terms of psychological distress and coping strategies with well-being (β = 0.078, p = 0.58) were found insignificant in statistical terms. These findings have implications for organizations and individuals in terms of identifying effective coping strategies to mitigate the detrimental effects of psychological distress on work-related outcomes i.e., individual work performance. This study is consistent with the result of Meister et al. (2023) [42] research. Their study demonstrated that negative affect and coping resource appraisals combined to influence the relationship between internal identity asymmetry and actual performance outcomes. The present study is the original to examine the positive interaction effect of coping strategies and psychological distress on an individual's work performance.

a. Theoretical Contribution

The results of the present investigation highlight several theoretical implications. First, we presented a novel perspective in identity literature concerning individuals' perceptions of others at work, an area that has been recognized as critical but understudied. Although self-awareness assists employees in predicting how their coworkers perceive them it does not investigate how people react to contradictory feedback from coworkers. Therefore, this study focuses on the psychological process that occurs when an individual believes they have been misidentified, such as internal identity asymmetry, which can have a negative impact on workplace attitudes.

Secondly, Employees may perceive positive and negative feelings during internal identity asymmetry. Researcher take these perceived negative feelings as Psychological distress in this study according to self-verification theory and model of stress & coping. The current study investigated this theory by empirically examining how psychological distress influences internal identity asymmetries' consequences on individual outcomes, i.e., Individual work performance (IWP) and well-being. It also developed a conceptual model that included psychological distress as a mediator between internal identity asymmetries and an individual's work performance (IWP) and well-being. The result indicates that employees face more psychological distress during negative base asymmetries. When there is more Psychological Distress it causes more effect on IWP & Well-being, whereas this is a novel contribution to internal identity asymmetry theory. Third, the present study examined the impact of internal identity asymmetry their behavioral and emotional outcomes through coping strategies by empirical testing. Findings suggest that the psychological distress of employees is reduced by incorporating coping strategies during various stages of their careers and time. These findings highlight the importance of coping strategies in improving the performance and well-being of employees. Hence, we contributed these findings to internal identity asymmetry theory.

Lastly, this finding enhances contextual specific knowledge as well; the context of the present study is Pakistan. The characteristics of Pakistan are collectivism, a large power distance, and a high level of uncertainty avoidance. These factors largely explain organizational practices like nepotism, centralization, and corruption. For instance, first, employees identify the asymmetry or discrepancy that makes them uncomfortable, tense, and dissatisfied. If asymmetries are fixed and long like gender or age, then they should not consume their time and energy to resolve these fixed asymmetries as they can't be changed so they need to maintain it. They have to focus on those asymmetries that are flexible or can enhance like enhance abilities & skills, working patterns, and performance, and foster positive attitudes. They had to work on these skills and resolve these asymmetries after getting proper feedback from their seniors, peers, or subordinates.

b. Managerial Contribution

Managers and practitioners must give importance to employees' perceptions and feelings to get positive outcomes. For instance, when individuals perceive negative feelings during their asymmetry times, the manager may support them through counseling, and the manager may share their personal experience and guide them accordingly. Practitioners need to know about various coping strategies, i.e., solving, maintenance & endurance, which employees need to adopt in response to the positive and negative impact of asymmetries. Social support is an essential part of incorporating strategies such as a supervisor or peer support, juniors, who can help the individual to handle the negative asymmetry situation and sort it out peacefully. Employees should use 360° feedback tools that assess self-other agreement to reduce internal identity asymmetries. This experience assists people in recognizing their identity asymmetries and may lead to coaching on managing, reducing, and changing them. While these tools are typically used to assess competencies, abilities, and personality traits, it is also important to consider and be receptive to asymmetries in other aspects of a person's self-identity.

c. Limitation

We have assumed that these findings are consistent with developing countries and have similar behavior; however, it will be empirically verified first. Even with the contribution, this study has some limitations to be acknowledged. First, this study explores the impact of internal identity asymmetries, i.e., personal base asymmetries and professional base asymmetries, on the performance and well-being of employees. It is suggested to study the personal base asymmetries and professional base asymmetries separately with their outcomes; for instance, future studies can measure the impact of personal based asymmetries (gender or physical appearance) and professional bases asymmetry (promotion, opportunities) more deeply with their individual and organizational outcomes. This study can include characteristics of people who possess high or low personal-based asymmetry and the characteristics of people who possess high or low professional-based asymmetry. Also, it would be beneficial to study how people with high personal-based asymmetry tend to affect their psychological distress and how people with high professional-based asymmetry tend to affect their psychological distress. This will help both organizations and individuals to make them aware about the intensity and negative impact of asymmetries which can guide them toward strategies that need to be employed to handle this situation. Second, we can capture the individual's experience of personal and professional base asymmetry as a whole, but we are unable to identify which specific identity is experiencing this asymmetry. However, it would be interesting to investigate whether and to what extent the "type" of asymmetry affects their outcomes. For instance, a person's competence related (intelligence) or role based (parent or HR director) identities may elicit different responses and outcomes from an asymmetry experience than a person's demographic (such as gender or ethnicity) identities.

Future research could build on these findings by examining how relational quality, such as conflict and belonging perceptions moderate the effect of internal identity asymmetry on the outcomes of interest. In this study, we didn't measure specific identities or sets of identities under which individuals experienced misidentification. As individuals are more likely to observe and categorize others based on how they differ from the majority. In future research, demographic differences between workers would be interesting to examine. Women in male-dominated workplaces may be more likely to experience identity asymmetry due to their gender. Addressing this discrimination-based asymmetry may be more effective by addressing the perceiver's biased and often adverse views and helping the perceived build coping response. Lastly, this study has been conducted and examined in a south Asian context, i.e., Pakistan. Due to a lack of time & resources, the author cannot collect data from other regions. However, it is advisable for future researchers to enhance the robustness of their findings by comparing them with data collected from diverse regions. Subsequent studies could gather data from multiple areas across Asia, allowing for comparative analysis to derive meaningful insights and contributions.

## Data availability statement

The data cannot be published because of the copyright policy of the original data. Data will be made available on request.

## CRediT authorship contribution statement

**Rida Batool:** Writing – review & editing, Writing – original draft, Visualization, Resources, Project administration, Methodology, Investigation, Formal analysis, Data curation, Conceptualization. **Qingfeng Tian:** Writing – review & editing, Validation, Supervision, Resources, Project administration, Methodology. **Erhua Zhou:** Visualization, Validation, Supervision, Methodology, Conceptualization. **Najmul Hasan:** Writing – review & editing, Software, Methodology, Formal analysis.

## Declaration of competing interest

The authors declare that they have no known competing financial interests or personal relationships that could have appeared to influence the work reported in this paper.

## References

[1] Ashforth B.E., Schinoff B.S. (2016). Identity under construction: how individuals come to define themselves in organizations. Annual Review of Organizational Psychology and Organizational Behavior.

[2] Elsbach K.D. (2004). Interpreting workplace identities: the role of office décor. J. Organ. Behav..

[3] Swann Jr W.B., Johnson R.E., Bosson J.K. (2009). Identity negotiation at work. Res. Organ. Behav..

[4] Burke P.J., Stets J.E. (1999). Trust and commitment through self-verification. Soc. Psychol. Q..

[5] Grutterink H., Meister A. (2022). Thinking of you thinking of me:c an integrative review of meta‐perception in the workplace. J. Organ. Behav..

[6] Meister A., Jehn K.A., Thatcher S. (2012). Intl. Association for Conflict Management, IACM 25th Annual Conference.

[7] Meister A., Jehn K.A., Thatcher S.M.B. (2014). Feeling misidentified: the consequences of internal identity asymmetries for individuals at work. Acad. Manag. Rev..

[8] Peters T. (1997). The brand called you. Fast Co..

[9] Ulrich D., Smallwood N. (2007). Building a leadership brand. Harv. Bus. Rev..

[10] Kim K.Y. (2016). Multisource feedback, human capital, and the financial performance of organizations. J. Appl. Psychol..

[11] Ouyang K., Lam W., Wang W. (2015). Roles of gender and identification on abusive supervision and proactive behavior. Asia Pac. J. Manag..

[12] Hewlin P.F. (2003). And the award for best actor goes to: facades of conformity in organizational settings. Acad. Manag. Rev..

[13] Hewlin P.F. (2009). Wearing the cloak: antecedents and consequences of creating facades of conformity. J. Appl. Psychol..

[14] Oltmanns T.F. (2005). Meta-perception for pathological personality traits: do we know when others think that we are difficult?. Conscious. Cognit..

[15] Higgins E.T. (1987). Self-discrepancy: a theory relating self and affect. Psychol. Rev..

[16] Burns B.D., Vollmeyer R. (1998). Modeling the adversary and success in competition. J. Pers. Soc. Psychol..

[17] Schlenker B.R., Leary M.R. (1982). Social anxiety and self-presentation: a conceptualization and model. Psychol. Bull..

[18] Moussaid M. (2013). Social influence and the collective dynamics of opinion formation. PLoS One.

[19] Higgins E.T., Klein R., Strauman T. (1985). Self-concept discrepancy theory: a psychological model for distinguishing among different aspects of depression and anxiety. Soc. Cognit..

[20] Bruch M.A., Rivet K.M., Laurenti H.J. (2000). Type of self-discrepancy and relationships to components of the tripartite model of emotional distress. Pers. Indiv. Differ..

[21] Phillips A.G., Silvia P.J. (2005). Self-awareness and the emotional consequences of self-discrepancies. Pers. Soc. Psychol. Bull..

[22] Homan A.C. (2007). Bridging faultlines by valuing diversity: diversity beliefs, information elaboration, and performance in diverse work groups. J. Appl. Psychol..

[23] Jehn K.A., Rispens S., Thatcher S.M.B. (2010). The effects of conflict asymmetry on work group and individual outcomes. Acad. Manag. J..

[24] Kark R., Meister A., Peters K. (2022). Now you see me, now you don't: a conceptual model of the antecedents and consequences of leader impostorism. J. Manag..

[25] Swann Jr W.B., Rentfrow P.J., Guinn J.S., Leary M.R., Tangney J.J.P. (2003). Handbook of Self and Identity.

[26] Meister A., Sinclair A., Jehn K.A. (2017). Identities under scrutiny: how women leaders navigate feeling misidentified at work. Leader. Q..

[27] Folkman S. (1986). Dynamics of a stressful encounter: cognitive appraisal, coping, and encounter outcomes. J. Pers. Soc. Psychol..

[28] Bell E., Sinclair A. (2016). Bodies, sexualities and women leaders in popular culture: from spectacle to metapicture. Gender in Management: Int. J..

[29] Wang Y., Kim Y., Lau D.C. (2023). Creative identity asymmetry: when and how it impacts psychological strain and creative performance. Asia Pac. J. Manag..

[30] Puranik H. (2019). They want what I’ve got (I think): the causes and consequences of attributing coworker behavior to envy. Acad. Manag. Rev..

[31] Karelaia N., Guillén L. (2014). Me, a woman and a leader: positive social identity and identity conflict. Organ. Behav. Hum. Decis. Process..

[32] Ramarajan L. (2014). Past, present and future research on multiple identities: toward an intrapersonal network approach. Acad. Manag. Ann..

[33] Vough H.C. (2013). What clients don't get about my profession: a model of perceived role-based image discrepancies. Acad. Manag. J..

[34] Ostroff C., Atwater L.E., Feinberg B.J. (2004). Understanding self‐other agreement: a look at rater and ratee characteristics, context, and outcomes. Person. Psychol..

[35] Strauss A., Corbin J. (1998). Basics of Qualitative Research: Techniques and Procedures for Developing Grounded Theory.

[36] Hofstede Insights (2021). Hofstede cultural dimensions. https://www.hofstede-insights.com/country-comparison-tool?countries=pakistan.

[37] Abbas M. (2012). Combined effects of perceived politics and psychological capital on job satisfaction, turnover intentions, and performance. J. Manag..

[38] Pfundmair M. (2015). The different behavioral intentions of collectivists and individualists in response to social exclusion. Pers. Soc. Psychol. Bull..

[39] Barsoum G. (2016). The public sector as the employer of choice among youth in Egypt: the relevance of public service motivation theory. Int. J. Publ. Adm..

[40] Jahanzeb S., Fatima T., Malik M.A.R. (2018). Supervisor ostracism and defensive silence: a differential needs approach. Eur. J. Work. Organ. Psychol..

[41] Batool R. (2019). Women leadership and their experience of internal identity asymmetry at workplace. International Journal of Research in Business and Social Science (2147-4478).

[42] Meister A. (2023). How feeling misidentified can drive negative attitudes yet increase performance: the role of appraisals. J. Appl. Soc. Psychol..

[43] Abelson R.P. (1968). Theories of Cognitive Consistency: a Sourcebook.

[44] Aronson E. (1969). Advances in Experimental Social Psychology.

[45] Hinojosa A.S. (2017). A review of cognitive dissonance theory in management research: opportunities for further development. J. Manag..

[46] Fournier M.A. (2015). Toward a unified science of personality coherence. Can. Psychol./Psychol. Canad..

[47] Ibarra H. (2015). The authenticity paradox. Harv. Bus. Rev..

[48] Lazarus R.S., Folkman S. (1984).

[49] Barreto M., Ellemers N. (2003). The effects of being categorised: the interplay between internal and external social identities. Eur. Rev. Soc. Psychol..

[50] Barreto M. (2010). To be or not to be: the impact of implicit versus explicit inappropriate social categorizations on the self. Br. J. Soc. Psychol..

[51] Roberts L.M. (2005). Changing faces: professional image construction in diverse organizational settings. Acad. Manag. Rev..

[52] Polzer J.T., Milton L.P., Swarm W.B. (2016). Capitalizing on diversity: interpersonal congruence in small work groups. Adm. Sci. Q..

[53] Thatcher S.M., Greer L.L. (2008). Does it really matter if you recognize who I am? The implications of identity comprehension for individuals in work teams. J. Manag..

[54] Swann Jr W.B., Milton L.P., Polzer J.T. (2000). Should we create a niche or fall in line? Identity negotiation and small group effectiveness. J. Pers. Soc. Psychol..

[55] Aguinis H., Kraiger K. (2009). Benefits of training and development for individuals and teams, organizations, and society. Annu. Rev. Psychol..

[56] Koopmans L. (2014). Improving the individual work performance questionnaire using rasch analysis. J. Appl. Meas..

[57] Koopmans L. (2014). Measuring individual work performance: identifying and selecting indicators. Work.

[58] Meunier S. (2022). The association between perceived stress, psychological distress, and job performance during the COVID-19 pandemic: the buffering role of health-promoting management practices. 2022: Trends in Psychol.

[59] Folkman S., Lazarus R.S. (1985). If it changes it must be a process: study of emotion and coping during three stages of a college examination. J. Pers. Soc. Psychol..

[60] Swann W.B. (1983). Social Psychology Perspectives.

[61] Swann W.B., Hill C.A. (1982). When our identities are mistaken: reaffirming self-conceptions through social interaction. J. Pers. Soc. Psychol..

[62] Smith C.A., Kirby L.D. (2009). Putting appraisal in context: toward a relational model of appraisal and emotion. Cognit. Emot..

[63] Zhou H. (2017). Mediating effect of coping styles on the association between psychological capital and psychological distress among Chinese nurses: a cross‐sectional study. J. Psychiatr. Ment. Health Nurs..

[64] Folkman S., Gellman M.D., Turner J.R. (2013). Encyclopedia of Behavioral Medicine.

[65] Koopmans L. (2014). Measuring individual work performance: identifying and selecting indicators. Work.

[66] Fisher C.D. (2010). Happiness at work. Int. J. Manag. Rev..

[67] Taheri F., Jami Pour M., Asarian M. (2019). An exploratory study of subjective well-being in organizations–A mixed method research approach. J. Hum. Behav. Soc. Environ..

[68] Creswell J.W., Creswell J.D. (2017).

[69] Raja U., Javed Y., Abbas M. (2018). A time lagged study of burnout as a mediator in the relationship between workplace bullying and work–family conflict. Int. J. Stress Manag..

[70] Cohen L.L., Swim J.K. (1995). The differential impact of gender ratios on women and men: tokenism, self-confidence, and expectations. Pers. Soc. Psychol. Bull..

[71] Sykes B.L., Verma A., Hancock B.H. (2018). Aligning sampling and case selection in quantitative-qualitative research designs: establishing generalizability limits in mixed-method studies. Ethnography.

[72] Yarkoni T. (2022). The generalizability crisis. Behav. Brain Sci..

[73] Ringle C.M., Wende S., Becker J.-M. (2022). SmartPLS 4. Oststeinbek: SmartPLS. http://www.smartpls.com.

[74] Hair Jr J.F. (2017). PLS-SEM or CB-SEM: updated guidelines on which method to use. International Journal of Multivariate Data Analysis.

[75] Williams D.R. (1997). Racial differences in physical and mental health: socio-economic status, stress and discrimination. J. Health Psychol..

[76] Slopen N. (2012). Psychosocial stressors and cigarette smoking among African American adults in midlife. Nicotine Tob. Res..

[77] Settles I.H. (2004). When multiple identities interfere: the role of identity centrality. Pers. Soc. Psychol. Bull..

[78] Essed P. (1991).

[79] Kessler R.C. (2002). Short screening scales to monitor population prevalences and trends in non-specific psychological distress. Psychol. Med..

[80] Furukawa T.A. (2008). The performance of the Japanese version of the K6 and K10 in the world mental health survey Japan. Int. J. Methods Psychiatr. Res..

[81] Carver C.S. (1997). You want to measure coping but your protocol's too long: consider the brief COPE. Int. J. Behav. Med..

[82] Jaworek M.A., Marek T., Karwowski W. (2020). The scale of work-related affective feelings (WORAF). Appl. Ergon..

[83] Van Katwyk P.T. (2000). Using the Job-Related Affective Well-Being Scale (JAWS) to investigate affective responses to work stressors. J. Occup. Health Psychol..

[84] Alalwan A.A., Dwivedi Y.K., Rana N.P. (2017). Factors influencing adoption of mobile banking by Jordanian bank customers: extending UTAUT2 with trust. Int. J. Inf. Manag..

[85] Hair J.F. (2010).

[86] Curran P.J., West S.G., Finch J.F. (1996). The robustness of test statistics to nonnormality and specification error in confirmatory factor analysis. Psychol. Methods.

[87] Podsakoff P.M. (2003). Common method biases in behavioral research: a critical review of the literature and recommended remedies. J. Appl. Psychol..

[88] Chang S.-J., van Witteloostuijn A., Eden L. (2010). From the Editors: common method variance in international business research. J. Int. Bus. Stud..

[89] Kock N. (2015). Common method bias in PLS-SEM. Int. J. e-Collaboration.

[90] Chou S.-W., Chang Y.-C. (2008). The implementation factors that influence the ERP (enterprise resource planning) benefits. Decis. Support Syst..

[91] Hair Jr J.F., Howard M.C., Nitzl C. (2020). Assessing measurement model quality in PLS-SEM using confirmatory composite analysis. J. Bus. Res..

[92] Hasan N. (2019). Factors affecting post-implementation success of enterprise resource planning systems: a perspective of business process performance. Enterprise Inf. Syst..

[93] Henseler J. (2014). Common beliefs and reality about PLS: comments on rönkkö and evermann (2013). Organ. Res. Methods.

[94] Henseler J., Ringle C.M., Sarstedt M. (2014). A new criterion for assessing discriminant validity in variance-based structural equation modeling. J. Acad. Market. Sci..

[95] Hair Jr J F. (2014). Partial least squares structural equation modeling (PLS-SEM). Eur. Bus. Rev..

[96] Cronbach L.J. (1951). Coefficient alpha and the internal structure of tests. Psychometrika.

[97] Nunnally J.C. (1978).

[98] Bagozzi R.P., Yi Y. (1988). On the evaluation of structural equation models. J. Acad. Market. Sci..

[99] Hair J.F. (2014). Partial least squares structural equation modeling (PLS-SEM). Eur. Bus. Rev..

[100] Chin W.W., Gopal A., Salisbury W.D. (1997). Advancing the theory of adaptive structuration: the development of a scale to measure faithfulness of appropriation. Inf. Syst. Res..

[101] Hair J.F. (2006).

[102] Fornell C., Larcker D.F. (1981). Evaluating structural equation models with unobservable variables and measurement error. J. Market. Res..

[103] Hair Jr J.F. (2016).

[104] Sarstedt M. (2014). On the emancipation of PLS-SEM: a commentary on rigdon (2012). Long. Range Plan..

[105] Rehman Khan S.A., Yu Z. (2021). Assessing the eco-environmental performance: an PLS-SEM approach with practice-based view. Int. J. Logist. Res. Appl..

[106] Li X. (2020). Family socioeconomic status and home-based parental involvement: a mediation analysis of parental attitudes and expectations. Child. Youth Serv. Rev..

[107] Rahi S. (2019). Integration of UTAUT model in internet banking adoption context. J. Res. Indian Med..

[108] Li J. (2023). How nursing students' risk perception affected their professional commitment during the COVID-19 pandemic: the mediating effects of negative emotions and moderating effects of psychological capital. Humanit Soc Sci Commun.

[109] Ellemers N., Spears R., Doosje B. (2002). Self and social identity. Annu. Rev. Psychol..

