# Supplementary material for: Impact of internal identity asymmetry on employee's behaviors and feelings: A mediating role of psychological distress
Source: Heliyon. 2024 May 17;10(10):e31438. doi: 10.1016/j.heliyon.2024.e31438 (PMC11130678; doi:10.1016/j.heliyon.2024.e31438)
Supplement: Multimedia component 1 [file mmc1.docx]

**Appendix A: Summary of measures of constructs, items & brief descriptions**

| **Construct** | **Item and a brief description** | **Sources of the Scale** |
| --- | --- | --- |
| **Personal based Asymmetry**  **(PBA)** | PBA1: Feel, being treated unfairly and get Misidentified at workplace.  PBA2: Feel being treated with less courtesy and respect than other people in daily work life.  PBA3: Think, not influential enough at workplace.  PBA4: Feel, other People think that not smart in daily work life.  PBA5: Feel that People act as if they’re better than me in daily work life  PBA6: Supervisor or coworkers make jokes about women at workplace. | [Williams et al. (1997)](#_ENREF_109)  [Settles (2004)](#_ENREF_93)  [Slopen et al. (2012)](#_ENREF_94) |
| **Professional based Asymmetry**  **(Pr.BA)** | Pr.BA1: Feel, being ignored or not taken seriously by Boss.  Pr.BA2: Feel, being ignored or not taken seriously by colleagues.  Pr.BA3: Feel, unfairly humiliated in front of others at workplace.  Pr.BA4: Coworkers with less experience and fewer qualifications get promotion before me.  Pr.BA5: Coworkers with less experience and fewer qualifications get Increment before me.  Pr.BA6: Require twice as much effort as others to receive the equal treatment and evaluation. | Chronic Work Discrimination and Harassment scale  ([Slopen et al., 2012](#_ENREF_94)) |
| **Psychological Distress**  (PD) | PD1: Often feel nervous.  PD2: Often feel hopeless.  PD3: Often feel nervous restless.  PD4: Constantly feel anxious, unhappy, and hopeless.  PD5: Often feel everything is a struggle.  PD6: Often feel unworthy. | Kessler Psychological Distress Scale (K6)  ([Kessler et al., 2002](#_ENREF_59)) |
| **Coping Strategies**  **(CS)** | CS1: Get advice or help from manager & colleague about what to do.  CS2: Focusing on changing the situation with my best efforts  CS3: Consider developing a plan of action.  CS4: Consider your options carefully before moving forward.  CS5: Making an effort to view it differently in order to make it appear more positively.  CS6: Seeking the positive aspects of what is happening.  CS7: Acknowledging that what has happened is real.  CS8: Saying to myself “this is not real.  CS9: Seeking other people's emotional support.  CS10: Seeking help and advice from other people.  CS11: Praying or meditating.  CS12: Doing something to think about less or other activities to take mind off.  CS13: Give up trying to deal with it.  CS14: Give up the attempt to cope. | Brief COPE Inventory designed [Carver (1997)](#_ENREF_16). |
| **Individual Work Performance**  **(IWP)** | IWP1: Manage to plan work, to done on time.  IWP2: Remember what you need to accomplish at work.  IWP3: Capable of distinguishing between major and minor issues at work.  IWP4: When previous tasks are complete, begin new ones by myself.  IWP5: Maintain job knowledge and skills by working to keep them current (IWP5).  IWP6: Take challenging work tasks, when available. | [Koopmans et al. (2014a)](#_ENREF_63)  [Koopmans et al. (2014b)](#_ENREF_64) |
| **Employee Wellbeing**  **(WB)** | WB1: Find, pleasurable work  WB2: A job can make you happy.  WB3: A job can make you feel fulfilled.  WB4: Discover fulfillment at work.  WB5: Happy with relations with superiors.  WB6: Positivity regarding the assignment and any difficulties at work  WB7: Overall feel, relaxed and free. | Work-Related Affective Feelings Scale (WORAF)  ([Jaworek et al., 2020](#_ENREF_54))  JAWS ([Van Katwyk et al., 2000](#_ENREF_106)) |

**Appendix B: Weights and loadings of the construct**

| Latent Variable | Items | Factor Loading | Standard Deviation | T Statistics | P Values | VIF | 2.5% | 97.5% |
| --- | --- | --- | --- | --- | --- | --- | --- | --- |
| Personal based Asymmetry (PBA) | PBA1 | 0.792 | 0.026 | 30.142 | 0.000 | 1.864 | 0.727 | 0.833 |
|  | PBA2 | 0.774 | 0.030 | 26.085 | 0.000 | 1.833 | 0.706 | 0.823 |
|  | PBA4 | 0.748 | 0.034 | 22.196 | 0.000 | 1.679 | 0.667 | 0.804 |
|  | PBA5 | 0.738 | 0.033 | 22.539 | 0.000 | 1.548 | 0.664 | 0.792 |
| Professional based Asymmetry  (Pr.BA) | Pr.BA1 | 0.773 | 0.027 | 28.359 | 0.000 | 1.932 | 0.706 | 0.817 |
|  | P. rBA2 | 0.758 | 0.029 | 26.327 | 0.000 | 1.898 | 0.695 | 0.808 |
|  | Pr.BA3 | 0.726 | 0.035 | 20.881 | 0.000 | 1.671 | 0.650 | 0.786 |
|  | Pr.BA4 | 0.823 | 0.024 | 34.607 | 0.000 | 3.125 | 0.767 | 0.861 |
|  | Pr.BA5 | 0.816 | 0.021 | 38.378 | 0.000 | 3.120 | 0.768 | 0.851 |
|  | Pr.BA6 | 0.743 | 0.030 | 24.835 | 0.000 | 1.629 | 0.674 | 0.793 |
| Psychologic al Distress  (PD) | PD2 | 0.827 | 0.020 | 41.499 | 0.000 | 2.012 | 0.781 | 0.861 |
|  | PD3 | 0.708 | 0.038 | 18.407 | 0.000 | 1.456 | 0.621 | 0.771 |
|  | PD4 | 0.834 | 0.019 | 44.447 | 0.000 | 1.886 | 0.791 | 0.865 |
|  | PD5 | 0.801 | 0.024 | 33.281 | 0.000 | 1.604 | 0.747 | 0.841 |
| Coping Strategies  (CS) | CS2 | 0.727 | 0.033 | 21.963 | 0.000 | 1.799 | 0.654 | 0.785 |
|  | CS3 | 0.844 | 0.017 | 48.412 | 0.000 | 2.422 | 0.806 | 0.875 |
|  | CS4 | 0.746 | 0.036 | 20.996 | 0.000 | 1.834 | 0.669 | 0.808 |
|  | CS5 | 0.783 | 0.028 | 28.093 | 0.000 | 2.022 | 0.720 | 0.830 |
|  | CS6 | 0.784 | 0.029 | 27.338 | 0.000 | 1.949 | 0.720 | 0.834 |
|  | CS7 | 0.733 | 0.033 | 21.952 | 0.000 | 1.561 | 0.657 | 0.789 |
| Individual Work Performance  (IWP) | IWP1 | 0.734 | 0.034 | 21.504 | 0.000 | 1.918 | 0.656 | 0.792 |
|  | IWP2 | 0.827 | 0.021 | 39.274 | 0.000 | 2.023 | 0.778 | 0.861 |
|  | IWP3 | 0.762 | 0.028 | 26.798 | 0.000 | 1.607 | 0.699 | 0.811 |
|  | IWP4 | 0.756 | 0.031 | 24.108 | 0.000 | 1.845 | 0.688 | 0.809 |
|  | IWP5 | 0.793 | 0.026 | 31.059 | 0.000 | 2.519 | 0.736 | 0.836 |
|  | IWP6 | 0.730 | 0.038 | 19.200 | 0.000 | 1.861 | 0.645 | 0.793 |
| Wellbeing  (WB) | WB1 | 0.800 | 0.024 | 33.345 | 0.000 | 2.147 | 0.749 | 0.841 |
|  | WB2 | 0.876 | 0.015 | 58.275 | 0.000 | 3.378 | 0.843 | 0.901 |
|  | WB3 | 0.852 | 0.017 | 49.598 | 0.000 | 3.160 | 0.815 | 0.882 |
|  | WB4 | 0.845 | 0.019 | 43.845 | 0.000 | 2.704 | 0.801 | 0.878 |
|  | WB5 | 0.710 | 0.033 | 21.326 | 0.000 | 1.667 | 0.638 | 0.769 |
|  | WB67 | 0.763 | 0.025 | 30.341 | 0.000 | 1.423 | 0.709 | 0.808 |

Note. VIF- Variance Inflation Factor, PBA − Personal based Asymmetry, Pr.BA − Professional based Asymmetry, PD − Psychological Distress, CS − Coping Strategies, IWP − Individual Work Performance, WB − Wellbeing.

**Appendix C: Survey Questionnaire**

I am Post doctorate fellow at Northwestern polytechnical University, Xian, China. I am conducting study on “Impact of Internal Identity Asymmetry on Employee’s Behaviors and Feelings: A Mediating role of Psychological Distress" for my research. There is often asymmetry between how we see ourselves, and how others actually see us. Internal identity asymmetry is experienced when individuals feel misidentified— when they believe their colleagues do not recognize their work-related identities. Your all information will be kept confidential and will be used only for research purpose. Thank you for your precious time and your support to complete this research study.

Regards

Dr. Rida Batool

**Section I: Demographic Information**

Please respond to the following questions by placing a **check mark (√)** in the answer box that corresponds to your response and or fill in the blank where indicated.

| Question | Response | | Code | | Response | | Code |
| --- | --- | --- | --- | --- | --- | --- | --- |
| Gender | Male | | 1 | | Female | | 2 |
| Age (years) | 20- 25 | | 1 | | 41 – 45 | | 5 |
|  | 26 - 30 | | 2 | | 46 – 50 | | 6 |
|  | 31 – 35 | | 3 | | >51 | | 7 |
|  | 36 – 40 | | 4 | |  | |  |
| Marital Status | Unmarried | | 1 | | Married | | 2 |
| Education | High School | | 1 | | Masters | | 3 |
|  | Bachelors | | 2 | | PhD | | 4 |
| Working Experience  (years) | 1-3 | | 1 | | 10-12 | | 4 |
|  | 4-6 | | 2 | | 13-15 | | 5 |
|  | 7-9 | | 3 | | > 16 | | 6 |
| Employment Status | Permanent | | 1 | | Contractual | | 2 |
| Career Level | Front | Officer/ Supervisor/Lecturer Assistant Manager/Executive | | | | | 1 |
|  | Middle | Manager/Sr. Executive /Assist. Professor/GM/ Country Manager/ Dy. Director/Professor | | | | | 2 |
|  | Top | COO/CFO/Dean/ CEO/Managing Director | | | | | 3 |
| Sector | Public | | | 1 | | Corporate | 4 |
|  | Semi Govt | | | 2 | | Multinational | 5 |
|  | Private | | | 3 | |  |  |
| Industry | Academia/Science | | | 1 | | Financial Services | 7 |
|  | Health Care | | | 2 | | Pharmaceutical | 8 |
|  | Manufacturing | | | 3 | | Technology | 9 |
|  | Telecommunications & Media | | | 4 | | Entertainment | 10 |
|  | Energy (Oil & Gas) | | | 5 | | Government | 11 |
|  | Non-Profit | | | 6 | | Other | 12 |

**Section II**

Please respond to the following questions by placing a **check mark (√)** in the answer box that corresponds to your response.

| Sr # | Misidentification at Workplace | Strongly  Disagree | Disagree | Neutral | Agree | Strongly  Agree |
| --- | --- | --- | --- | --- | --- | --- |
| 1 | I feel, I’m being treated unfairly and get Misidentified at workplace. | 5 | 4 | 3 | 2 | 1 |
| 2 | I feel that I am being treated with less courtesy and respect than other people in my daily work life. | 5 | 4 | 3 | 2 | 1 |
| 3 | I think that I am not influential enough at workplace. | 5 | 4 | 3 | 2 | 1 |
| 4 | I feel that other People think that I am not smart in daily work life. | 5 | 4 | 3 | 2 | 1 |
| 5 | I feel that People act as if they’re better than me in daily work life. | 5 | 4 | 3 | 2 | 1 |
| 6 | My supervisor or coworkers make jokes about women at workplace. | 5 | 4 | 3 | 2 | 1 |
| 7 | I feel that I am being ignored or not taken seriously by my Boss. | 5 | 4 | 3 | 2 | 1 |
| 8 | I feel that I am being ignored or not taken seriously by my colleagues. | 5 | 4 | 3 | 2 | 1 |
| 9 | I feel, I am unfairly humiliated in front of others at workplace. | 5 | 4 | 3 | 2 | 1 |
| 10 | My coworkers with less experience and fewer qualifications get promotion before me. | 5 | 4 | 3 | 2 | 1 |
| 11 | My coworkers with less experience and fewer qualifications get Increment before me? | 5 | 4 | 3 | 2 | 1 |
| 12 | I feel that I need to work twice as hard as others to get the same treatment or evaluation. | 5 | 4 | 3 | 2 | 1 |
| Psychological Distress | | All the time | Most of the time | Some of the time | Little of the time | None of the time |
| 1 | How often did you feel nervous? | 4 | 3 | 2 | 1 | 0 |
| 2 | How often did you feel hopeless? | 4 | 3 | 2 | 1 | 0 |
| 3 | How often did you feel restless? | 4 | 3 | 2 | 1 | 0 |
| 4 | How often did you feel so depressed that nothing could cheer you up? | 4 | 3 | 2 | 1 | 0 |
| 5 | How often did you feel that everything was an effort? | 4 | 3 | 2 | 1 | 0 |
| 6 | How often did you feel worthless? | 4 | 3 | 2 | 1 | 0 |
| Coping Strategy | | A Lot | Quite A Bit | Medium Amount | A  Little Bit | Not at All |
| 1 | I try to get advice or help from Manager/Colleague about what to do. | 5 | 4 | 3 | 2 | 1 |
| 2 | I am concentrating my efforts on doing something about the situation I'm in. | 5 | 4 | 3 | 2 | 1 |
| 3 | I try to come up with a strategy about what to do. | 5 | 4 | 3 | 2 | 1 |
| 4 | I think hard about what steps to take. | 5 | 4 | 3 | 2 | 1 |
| 5 | I am trying to see it in a different light, to make it seem more positive. | 5 | 4 | 3 | 2 | 1 |
| 6 | I am looking for something good in what is happening. | 5 | 4 | 3 | 2 | 1 |
| 7 | I am accepting the reality of the fact that it has happened. | 5 | 4 | 3 | 2 | 1 |
| 8 | I’m saying to myself “this is not real”. | 5 | 4 | 3 | 2 | 1 |
| 9 | I am getting emotional support from others. | 5 | 4 | 3 | 2 | 1 |
| 10 | I am getting help and advice from other people. | 5 | 4 | 3 | 2 | 1 |
| 11 | I am praying or meditating. | 5 | 4 | 3 | 2 | 1 |
| 12 | I am doing something to think about it less or other activities to take my mind off. | 5 | 4 | 3 | 2 | 1 |
| 13 | I'm giving up trying to deal with it. | 5 | 4 | 3 | 2 | 1 |
| 14 | I’ve given up the attempt to cope. | 5 | 4 | 3 | 2 | 1 |
| Individual Working Performance | | Always | Often | Frequently | Some-times | Seldom |
| 1 | I manage to plan my work so that is done on time. | 5 | 4 | 3 | 2 | 1 |
| 2 | I do keep in mind the results that I have to achieve in my work. | 5 | 4 | 3 | 2 | 1 |
| 3 | I am able to separate main issues from side issues at work. | 5 | 4 | 3 | 2 | 1 |
| 4 | I do start new tasks by myself, when my old ones are finished. | 5 | 4 | 3 | 2 | 1 |
| 5 | I do work at keeping my job KSA’s up-to-date. | 5 | 4 | 3 | 2 | 1 |
| 6 | I do take challenging work tasks, when available. | 5 | 4 | 3 | 2 | 1 |
|  | **Employee Wellbeing** | Ext.  Often | Often | Some-times | Rarely | Never |
| 1 | I find my work enjoyable. | 5 | 4 | 3 | 2 | 1 |
| 2 | My job brings me satisfaction. | 5 | 4 | 3 | 2 | 1 |
| 3 | My job gives me a sense of fulfillment. | 5 | 4 | 3 | 2 | 1 |
| 4 | I find contentment in my work. | 5 | 4 | 3 | 2 | 1 |
| 5 | I am happy with my relations with my superiors. | 5 | 4 | 3 | 2 | 1 |
| 6 | I have a positive attitude toward the task and problems which I am facing at work. | 5 | 4 | 3 | 2 | 1 |
| 7 | Overall, I feel relaxed and free. | 5 | 4 | 3 | 2 | 1 |
